# Using the stent-in-stent technique to drain the right hepatic duct, passing across a 7-year-old obstructed spiral self-expandable metal stent of the left hepatic duct

**DOI:** 10.1093/gastro/goaa019

**Published:** 2020-05-05

**Authors:** Ahmad Alhamid, Ziad Aljarad, Fayez Sandouk

**Affiliations:** 1 Internal Medicine, Faculty of Medicine, University of Aleppo, Aleppo, Syria; 2 Department of Gastroenterology, Aleppo University Hospital, Aleppo, Syria; 3 Gastroenterology Specialty Center, Damascus, Syria

## Introduction

Endoscopic stent implantation is a widely used and accepted palliative procedure for non-operated malignant hilar biliary obstruction [1]. Several studies have demonstrated that self-expandable metal stents (SEMS) are superior to plastic stents (PS) [2, 3]. However, it is still debated whether bilateral stenting is better than unilateral stenting [2, 3]. Park *et al.* reviewed the literature and clarified that bilateral stenting is favored, due to better clinical outcomes and fewer late complications [2]. It is technically challenging to place two SEMS beside each other in order to do bilateral stenting [1, 2]. For the above considerations, a new technique called ‘stent-in-stent’ (SIS) has been developed [1]. In this method, a Y-stent with a central open mesh allows a second stent to be introduced into the contralateral bile duct [1], resulting in a technical success ranging from 80% to 100% [2].

In this article, we report a SIS procedure, in which two SEMS were placed across each other. The second SEMS was introduced into the right hepatic duct, passing through an old, completely obstructed, non-removable SEMS that was draining the left hepatic duct 7 years ago. Notably, the old SEMS is an uncovered spiral stent, not a Y-stent designed especially for SIS. This procedure represents an alternative to the side-by-side stent placement if the old one is obstructed. Also, it allows the application of the SIS technique in previous patients whose biliary tree was primarily drained by unilateral stenting, especially those with obstructed and non-removable first SEMS. In addition, this case proves the possibility and reliability of using spiral SEMS for SIS if Y-stents are not available.

## Case presentation

A 60-year-old Syrian male swimmer suffered from non-resectable non-Hodgkin lymphoma in the head of the pancreas, which led to common bile duct (CBD) obstruction. It was first misdiagnosed as adenocarcinoma. We performed endoscopic retrograde cholangiopancreatography (ERCP) and placed an uncovered spiral SEMS to drain the left hepatic duct, and put the patient on chemotherapy. We placed the uncovered stent (WallFlex Biliary RX Uncovered Stent System RMV) because the tumor was believed to be adenocarcinoma.

Two years later, the patient developed ascending cholangitis. The ERCP revealed a highly localized, completely obstructed metal stent with ingrowing tissue, with a solidified smudge inside and around it. We could not remove the metal stent or insert the guide wire into it. We performed magnetic resonance cholangiopancreatography (MRCP) and placed an internal–external biliary-drainage catheter by percutaneous transhepatic cholangiography. The external–internal biliary-drainage catheter was changed every 6 months. Four years later, we removed the biliary-drainage catheter because the tumor regressed and the CBD obstruction cured.

**Figure 1.**
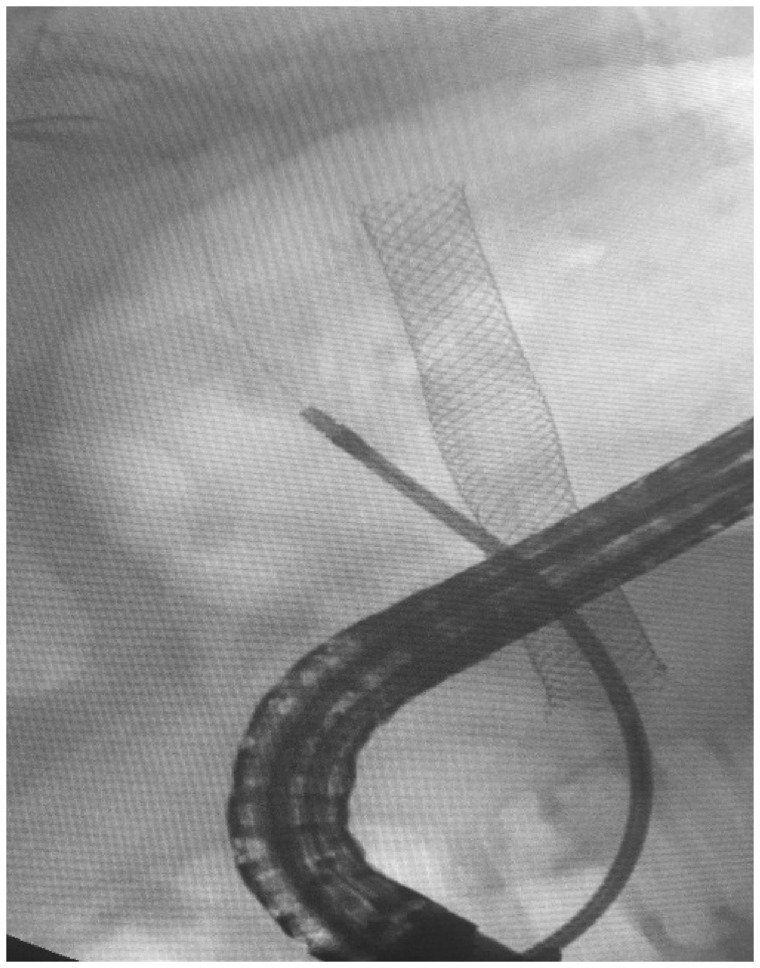
Insertion of a Soehendra dilator over the guide wire into the right hepatic duct

**Figure 2.**
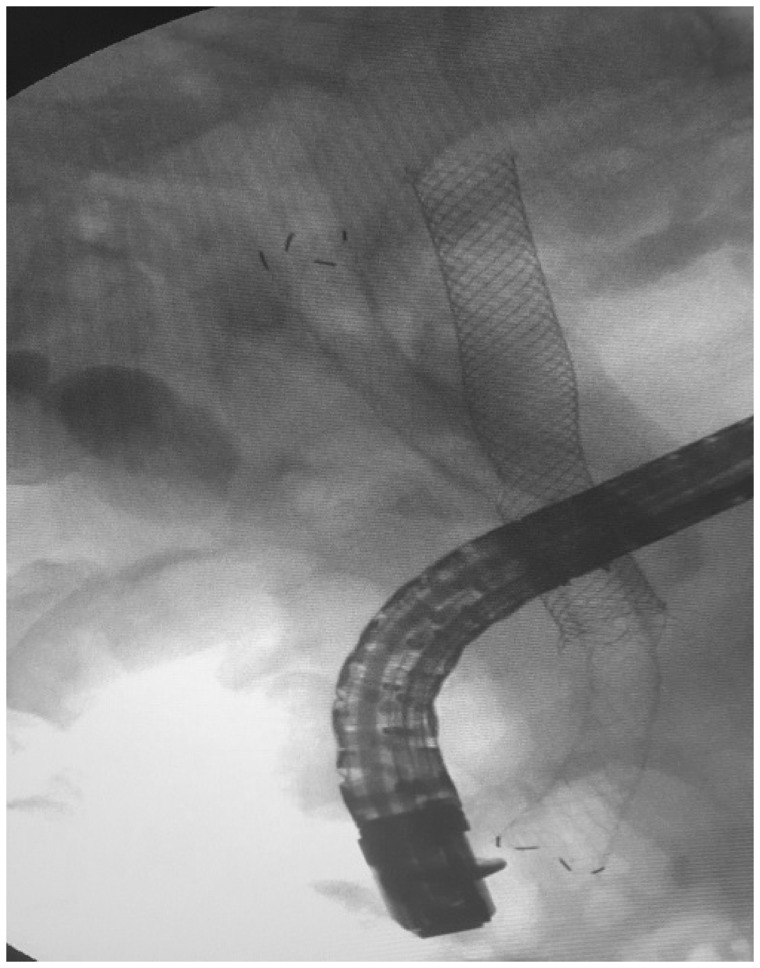
Insertion of a fully covered metal SEMS into the right hepatic duct through the uncovered metal SEMS, Y-shaped in appearance

After 1 year, the biliary obstruction recurred. The complete obstruction of the metal SEMS resulted in fibrosis of the left hepatic lobe. The right hepatic duct was dilated. Surgeons suggested lower CBD resection with right choledocojejunostomy or liver transplant. We wanted to give it a last try with ERCP before resorting to surgery. We could insert a guide wire through the obstructed SEMS into the right hepatic duct. Then, we inserted a Sohendra dilator (Soehendra^®^; size: 5 French; length: 180 cm; wire guide diameter: 0.021 inch) over the guide wire into the right hepatic duct (Figure 1). After that, we performed a trans-SEMS balloon dilation. Finally, we placed a fully covered metal SEMS (Hanaro^®^ Fully Covered Self-Expandable Metal Stents with New ‘Anchoring Flap’ System) into the right hepatic duct through the previously uncovered metal SEMS, to get two SEMS across each other. This gave us a Y-shaped appearance (Figure 2). The whole procedure took 75 minutes. The patient is still under follow-up after 3 years since the last procedure. He is in good health and has had no biliary colic or ascending cholangitis.

## Discussion

In this article, we reported the placement of a new, metal, fully covered SEMS, passing through an old, obstructed, metal, uncovered SEMS. We consequently had two SEMS across each other, having a Y-shaped appearance. We did this procedure in order to drain the right hepatic duct in a CBD obstruction case, because the stent that was draining the left one was completely obstructed.

Lee *et al.* developed the SIS technique in order to drain the biliary tree bilaterally, avoiding the difficulties and challenges of side-by-side stents placement [1]. In this technique, we insert a SEMS through a Y-stent, which allows the drainage of both hepatic ducts. The Y-stent is provided with a central open mesh, allowing the second SEMS to pass through it towards the contralateral hepatic duct. However, in our case, the two stents were not placed in the same session; they were 7 years apart. In addition, the first stent was obstructed, non-removable, and a spiral stent, not a Y-stent.

Our approach represents an alternative to the side-by-side stent placement if the old one is obstructed. In addition, our procedure allows endoscopists to apply the SIS technique years after the old stent implantation in order to drain the biliary tree bilaterally, even though the old stent is not a Y-stent. This will help to reduce the biliary re-obstruction rate as well as the need for surgery. To our knowledge, this is the first report of a new SEMS placement through an 8-year-old obstructed one.

Our procedure has limitations. It was difficult and time-consuming to insert the guide wire and the Sohendra dilator through the ingrowing tissue and the solidified smudge, and we failed to insert the guide wire years ago when we first discovered the obstruction. This may indicate a high failure rate of the procedure and the need for an expert hand and a long time. In addition, we still do not know the consequences of placement of a SEMS through the pores of a spiral stent, which are not wide like the central pores of a Y-stent. However, the 2-year follow-up outcomes of our case are promising. We recommend further trials to investigate the success rate and follow-up outcomes of this procedure.
